# The Graduate Nurse Yesterday, Today, and Tomorrow1Address delivered at the commencement exercises of the Brooks Memorial Hospital, Dunkirk, N. Y., May 12, 1095.

**Published:** 1905-07

**Authors:** F. Park Lewis

**Affiliations:** Buffalo, N. Y.


### The Graduate Nurse Yesterday, Today, and Tomorrow.[1]

By F. PARK LEWIS, M. D., Buffalo, N. Y.

IT IS a pleasure to unite with you this evening in the exercises which complete the year of your hospital training. No one, perhaps, who has not been actively engaged in work of this character can have an adequate realisation of its full significance. No one who has not actually experienced it can know how much it means to endure the hard discipline of hospital work, with its long hours, its rigid requirements and its drudgery, for the woman who has chosen for herself one of the finest, one of the most noble, as it is one of the most womanly occupations possible to her, that of the trained nurse.

In her laborious hours of preparation she must master an unfamiliar nomenclature, she must conquer the difficulties of modern asepsis, she must school herself to stand by the surgeon without growing faint; she must learn to govern her tongue, to control her temper, to be quiet, to be ready, to be dextrous,—to have the qualities of a soldier while retaining the heart and sensibilities of a woman. All this she must learn besides the technical points of the work she has undertaken, and it takes character and courage to plan such a career and successfully carry it through. Therefore, I congratulate you, young women, members of this graduating class, each of whom must possess in no small degree the quality of initiative which Hugo Munsterberg says is the distinctive trait of American character.

I know, also, something of the thought and effort that anticipated the establishment of your training school. I know something of the evenings devoted to meetings for the consideration of plans and projects which resulted in that which made your education possible; the study of ways and means to meet the added burden which such work always involves. I congratulate, therefore, your training school committee in that they are able to present to the world so admirable a product of their labors tonight.

But this is not all. The demands made daily upon the time and energy of the busy physician make it no easy task for him to lecture before a school, and much that is best of brain and strength goes into the fitting a class for its life work. Hence, I congratulate the teaching faculty in sending out this class of trained women to help in the world's work; since, arduous though their task may sometimes have seemed, they are getting

1. Address delivered at the commencement exercises of the Brooks Memorial Hospital, Dunkirk, N. Y., May 12, 1095.

a larger reward than would result from the performance of their own especial duties; for while the physician by his skill helps the individual, as a teacher he sends out a class who help multitudes and the benefit is ever in progressive ratio.

I am aware that it is customary for the speaker who addresses a class of graduates to devote the hour to wholesome suggestions as to the manner of conduct after the tether has been broken and they have been set free to live independent, professional lives; but I doubt not that after two years spent within the hospital walls under the school discipline, guided by precept as well as by practice in daily hospital work, any further word of mine on either the etiquette or the ethics of nursing would be superfluous. I will not assume the office of mentor, therefore, but it may not be without interest, and perhaps of some practical value for all of us if we put ourselves outside of the immediate present and, looking through the vista of years past and to come, we consider the evolution, the present status and the future of the trained nurse; in other words, the

GRADUATE NURSE YESTERDAY, TODAY, AND TOMORROW.

So intimately are we all a part of the current of modern life that we lose our perspective, and find difficulty in assigning a proper place in history to momentous movements because we are in and of them. It is difficult for us to realise that at no time in the world's history have tremendous epoch-making events followed in such rapid succession as at the end of the nineteenth and the beginning of this twentieth century. The past quarter century has seen the invention of many things which we already regard as necessities, and the perfection of many more.

It has seen the proving and general acceptance as fact of much that existed only as theory and in the minds of a few great men. It has seen in the vast increase of books and periodicals, of schools and colleges, of transit and communication, of system and coöperation, the establishment of an absolutely new era, an absolutely new standard, an absolutely new possibility for every man and woman living today. Through the coöperation of our cable post and telegraph systems even dwellers in remote country places are put in direct touch with the news of two continents, with the most recent thought in literature, with the latest researches in science. And we have a new world of science.

We predicate with accuracy and precision the existence of worlds beyond the range of the telescope, we anticipate the discovery of a new element and put it in its proper place in harmony with demonstrated law before it has ever been seen by human eye.

The bacteriologist proves to us that whole groups of diseases are dependent upon certain low forms of vegetation, microscopic in character, finding lodgment in the vulnerable animal body. The biologist enunciates the truth that spontaneous generation of living forms is impossible, that without preëxisting life there can be no life, and lo, the whole world of preventive medicine and hygiene is opened to us.

The chemist, from his researches in the laboratory analyses and synthecises until he is able to give us the refinement of the drug freed from its inert or worthless properties. The surgeon applies it together with the principles of surgical cleanliness and his death rate is reversed. He saves 95 to 99 per cent. even where formerly the inevitable death rate was enormous.

We now realise that almost better than the cure of disease is its prevention, and in this we are helped by the ceaseless efforts of the specialists in physiology who study the bolus of food from each step of its preparation, its entry into the stomach, its reception by the peptic juices, its passage through the pancreatic and biliary secretions until, emulsified, it passes into the blood current and becomes the final brain cell, the organ of the mind. To the waiting world the physicist gives the result of his labors, and his most advanced studies in electrophysics are utilised in the form of radiotherapy.

All of these constitute the armamentarium of the modern physician. We see, then, that he is an evolutionary product; that he is able to make constant, daily use of the facts unknown a quarter of a century ago, of aids and of instruments invented and perfected within our own lifetime, of the results of experiments made in all quarters of the globe almost as soon as they are completed. It may be said that for him the "point that was yesterday invisible is his goal today and his starting point tomorrow." As his own work has changed in character and increased in scientific accuracy his demands have grown more exacting.

The good dame who used to make flaxseed poultices and draughts of sage tea, could give no adequate assistance to the physician or surgeon of today. The modern trained nurse, meeting her larger responsibilities and her more exacting and important duties, as the physician meets his, is no less an evolutionary product than he. She is an answer to necessity, she is a sum of effort; but as the physician rises each day to gaze upon an ever widening horizon, so also must she.

THE GRADUATE NURSE AN EVOLUTIONARY PRODUCT.

As the physician has during the past few years drawn upon constantly multiplying and extending sources for his data and his instruments, so has the woman who has grown to be, to such a phenomenal degree, his assistant and colaborer. But though his aims and her efforts lie along the same line, they are not identical and while much that he seeks is necessary to her also, each has requirements beyond those of the other. The trained nurse, as well as the physician, must understand that invisible pathogenic germs are more formidable than lions in the path and to do this she must have some conception of the principles of bacteriology, for it is she who makes surgically clean the operating room, sterilises the dressings and who strives to purify not merely the clothing but the atmosphere of the patient insolated with contagious disease.

She must know the meaning of the zig-zag temperature looking like saw-teeth on the daily chart, for general sepsis must have immediate recognition. She must be familiar with the details of the diet kitchen, for here is often a path where the physician fails to follow, and the directions which she receives are too frequently glittering generalities, the details of which she must herself supply. Where the duties of a trained nurse make her necessities and her knowledge different from that of the physician she has had, and will have, doubtless, to recognise these necessities and to plan ways and means of securing them.

It was through the efforts of nurses themselves that a uniform standard of admission to the profession of trained nursing was secured. Until 1903 there were absolutely no restrictions in the state of New York except those which each hospital adopted for itself. Now, in addition to a preliminary common school education, the law provides that any candidate for the practice of nursing must be not less than 21 years of age, of good moral character, have had at least two years of instruction and received a diploma from a training school maintaining a standard satisfactory to the regents. Although the practice of trained nursing goes back in this country as far as the year 1873, when three schools were established almost simultaneously in New York city, Boston, and New Haven, it is only within the last dozen years that the training school has become practically a feature of hospitals everywhere throughout the country.

At the risk of being too diffuse I wish to give you a few statistics, which will perhaps prove as surprising to you as they were to me. To illustrate the growth of your profession in this country, I quote from the reports of the United States Commissioner of Education. In 1880, the number of pupil nurses was 323; in 1890 there were 1,552; in 1900, 11,164; and in 1903, 13,779.

In greater New York there are now almost as many pupil nurses enrolled as five years ago were to be found in the entire United States. There are eleven training schools in the city of Buffalo alone, exclusive of that connected with the State Hospital. There is one connected with Memorial Hospital at Niagara Falls and one with the Memorial Hospital at Dunkirk, before whose graduating class I have the honor of speaking tonight, one at Jamestown, one at Elmira, one at Corning, one at Canandaigua and others at other towns in Western New York, while the central and eastern parts of the state are in no degree behind. The city of Rochester has six schools with nearly 200 pupils, and a like center is found in Syracuse and another in Albany.

But this growth, gourd like in its rapidity, has not yet had time to assume an orderly development. With this multitude of schools there is as yet

NO UNIFORMITY OF CURRICULUM.

In some, one lecture is given weekly, in others a complete and thorough course is systematically carried out. In some, the study of household economics forms an important, not to say fundamental, part of the course; in others there are few or no arrangements making this possible. In some, the most thorough pedagogical methods are pursued, each day's work and each week's lecture being the result of previous work and leading to the next. In others, each lecturer is free to choose his own subject, and it is not necessarily connected with that which preceded or those to follow it.

This lack of uniformity is a natural, and perhaps inevitable part of evolution; but if trained nursing is to become an integral part of the educational system of the state, so chaotic a condition of affairs should not be tolerated longer than is positively necessary. Schools connected with the larger and better equipped hospitals have certain advantages which make it difficult to emulate them, but better system and a nearer approach to uniform methods is certainly possible.

To this end each one of you graduate nurses must work, each one of us physicians, each one of you large hearted, helpful and progressive citizens, who have striven to make your training school what it is, must continue to strive to make it and all other schools better by your interest, your understanding and, whenever may be, your efforts. As we have just said the larger institutions with their more complete paraphernalia have been able to attain a degree of proficiency that is alternately discouraging and inspiring. But since it is only by aiming at the highest that we can hope to reach the best achievements possible to us, let us look for a moment at some of the methods of one of the best training schools in the country, that connected with Johns Hopkins University in Baltimore. The Hospital School is part of the Johns Hopkins Hospital and is under the same government. The school buildings are separated from the hospital buildings however, and the instruction in the properties and preparation of foods and in their application to the needs of the sick is given in a model kitchen equipped for teaching purposes.

The plan of instruction for the first six months is given to a study of household economics, hygiene and sanitation, anatomy, physiology, materia medica, and elementary nursing. This is considered a probationary period, and is designed to give the student some of the fundamental principles of nursing before she is brought into contact with patients. Those who give satisfactory evidence of a general fitness for the work of nursing are then placed in the wards of the hospital for further work and study. Teaching is given in the operating rooms and other clinics as well as at the school. The hospital has 360 beds, so that a large practical experience is afforded the student nurses. The faculty is large, there being four assistant superintendents, two instructors in practical nursing, two in dietetics, one instructor in massage, ten head nurses and assistant instructors (all of them women) besides the physicians.

During the first year students are instructed in marketing, the cost and care of foods as well as cooking; the care of kitchens, pantries, utensils, fuel, and the like, as well as especial training in the preparation of food for infants and for the sick. A study of plumbing and drainage, the principles involved and care necessary to maintain healthful conditions is included, and also a careful study of laundries and laundry work and of linen room and the care and distribution of linen, as well as of bedrooms and surgical wards. The three years' course required in the Johns Hopkins school makes possible all this preliminary work; and it gives to its graduate nurses a training almost equal to that of the Drexel Institute in domestic science, the Boston Cooking School and the Philadelphia School of Massage in their respective branches.

It may be objected that so elaborate a curriculum and so long a course of study would not be possible to all young women otherwise fitted to undertake this important profession, and even if it were possible to attain something approaching uniformity in the various training schools throughout the country, would it be desirable to try to place the standard so high? To this I would answer: by all means put the standard as high as possible, but if necessary have two grades of nurses. As there are many nurses who could not give time to so complete a course of study, so there are patients who are not ill enough to require, or who lack the means to employ the highest priced nursing. Let there be two distinct grades then, those able to pursue the longer and more elaborate course of study, getting, as they should, to compensate for the larger outlay, a larger wage. But the long course should, so far as is possible, be in every school the same, and the short course uniform as well.

Now the only way in which this seems to me to be even approximately possible is through a plan which has occurred to me, which appeals strongly to my imagination and which would make possible such splendid advantages that it would surely seem worth the effort it would cost to try it. The idea is one which underlies all present day progress, one which is fundamental in all great business enterprises is, indeed, in everything the spirit of the times, the watch word of the future—coöperation.

The fact that what is impossible to one may be easily attained by a dozen is being proved, if proof were needed, at this moment, in all quarters of the globe, in almost every department of human activity. Why not prove it again in solving the difficult problem of how to establish a uniform and high standard in our training schools, a problem which we find so hopelessly "too much" for any one to grapple with? I would like to see established at various centers throughout the state what might be termed the

### UNIVERSITY TRAINING SCHOOL.

Let the various hospitals appoint a member from each of their respective faculties to confer with members of the state universities, and decide upon a uniform course of instruction which could be carried on in the different hospitals throughout the state. Any course or courses requiring paraphenalia not usual in a hospital, or for any reason not possible to give in the majority of the hospitals, might be arranged for at the universities; part of the work being thus concentrated could be better given, at less expense under pedagogical methods, while much of it could be done, as it now is, in each hospital, to its own nurses, but this work being planned by a committee from each hospital staff joining the faculty of the university would be uniform and connected. This would, among other advantages too obvious to need pointing out, give a young woman from any one of these institutions holding the university diploma, a distinct standing known and acknowledged throughout the state.

Fuller courses in anatomy and physiology could be given in the universities than are usually possible in most of our training schools, and courses in sanitation, hygiene, and domestic economy established by the various universities might not be limited to the training schools' pupils, since a sufficient number of students to make the venture pay for itself would probably be glad to take advantage of the opportunity offered if thrown open to the public.

I cannot, however, take your time tonight to enter into any further details. The advantages and disadvantages of the plan would have to be discussed by the faculties of the hospitals and members of the staffs of the different hospitals with the training school committees. It seems to me that the advantages are beyond computation and the disadvantages certainly not greater than lie in the path of all progress. If it is worth while to raise your profession to a higher level, to bring order and power out of the inevitable chaos of its comparative incipiency, it is worth some effort and some sacrifice to bring this about. It was through the effort of graduate nurses that a beginning has been made toward lifting your profession to the place it should hold—second to none that any woman may occupy. It remains for you and those who work with you to further and fulfil this ideal.

454 FRANKLIN STREET.

